# Performance of a safe and dignified burial intervention during an Ebola epidemic in the eastern Democratic Republic of the Congo, 2018–2019

**DOI:** 10.1186/s12916-023-03194-x

**Published:** 2023-12-05

**Authors:** Abdihamid Warsame, Gwendolen Eamer, Alaria Kai, Lucia Robles Dios, Hana Rohan, Patrick Keating, Jacques Katshishi, Francesco Checchi

**Affiliations:** 1 Faculty of Epidemiology and Population Health, London School of Hygiene & Tropical Medicine, London, UK; 2 International Federation of Red Cross and Red Crescent Societies, Geneva, Switzerland; 3 Faculty of Public Health and Policy, London School of Hygiene & Tropical Medicine, London, UK; 4 UK Public Health Rapid Support Team, London School of Hygiene & Tropical Medicine, London, UK; 5 Red Cross Society of the Democratic Republic of Congo, Kinshasa, Democratic Republic of the Congo

**Keywords:** Ebola, Epidemic, Burial, Dead body, Performance, Intervention, Evaluation, Democratic Republic of Congo

## Abstract

**Background:**

A protracted Ebola Virus Disease (EVD) epidemic in the eastern Ituri, North and South Kivu provinces of the Democratic Republic of Congo (DRC) caused 3470 confirmed and probable cases between July 2018 and April 2020. During the epidemic, the International Federation of Red Cross and Red Crescent Societies (IFRC) supported the DRC Red Cross and other local actors to offer safe and dignified burials (SDB) for suspected and confirmed EVD cases, so as to reduce transmission associated with infectious dead bodies. We conducted a retrospective cohort study of the SDB service’s performance in order to inform future applications of this intervention.

**Methods:**

We analysed data on individual SDB responses to quantify performance based on key indicators and against pre-specified service standards. Specifically, we defined SDB timeliness as response within 24 h and success as all components of the service being implemented. Combining the database with other information sources, we also fit generalised linear mixed binomial models to explore factors associated with unsuccessful SDB.

**Results:**

Out of 14,624 requests for SDB, 99% were responded to, 89% within 24 h. Overall, 61% of SDBs were successful, somewhat below target (80%), with failures clustered during a high-insecurity period. Factors associated with increased odds of unsuccessful SDB included reported community and/or family nonacceptance, insecurity and suspensions of the EVD response, low health facility coverage and high coverage of radio and telephony. Burials supported by mobile Civil Protection (local authorities) and/or static, community-based ‘harm reduction’ teams were associated with lower odds of failure.

**Conclusions:**

A large-scale, timely and moderately performant SDB service proved feasible during the challenging eastern DRC EVD response. Burial teams that are managed by community actors and operate locally, and supported rather than owned by the Red Cross or other humanitarian organisations, are a promising modality of delivering this pillar of EVD control.

**Supplementary Information:**

The online version contains supplementary material available at 10.1186/s12916-023-03194-x.

## Background

Between July 2018 and April 2020, the Democratic Republic of the Congo (DRC) experienced its tenth recognised Ebola Virus Disease (EVD) epidemic [1], affecting the eastern provinces of North Kivu, Ituri and South Kivu and causing a total 3470 confirmed and probable recorded cases; of this total, 2287 (66%) were fatal [2]. Compared to the start of the 2013–2015 West Africa epidemic, the DRC response benefited from improved knowledge on EVD and the availability of vaccines and multi-drug treatments [3]. Despite this, the epidemic proved difficult to control. Response efforts were hampered by insecurity, political tensions, misinformation and public distrust [4, 5]. The response itself was criticised as not inclusive and insufficiently responsive to community feedback and the lived experience of populations affected [6–8].

Transmission of Ebola virus (EBOV) occurs via direct physical contact with an infected person, infected body fluids or contaminated fomites [9, 10]. Corpses of EVD cases are particularly infectious due to high viral loads and EBOV remaining viable for days after death [11]. Post-mortem care and practices involving contact with the deceased, such as preparation of the body (washing, cleansing, dressing), funerary rites (touching) and burial (placing of the body in a coffin and/or grave), are thus high-risk. The super-spreading role of funerary and burial practices was documented during the West Africa epidemic [12–15]. In DRC, as elsewhere, these practices are customarily performed by family members and the local community [16, 17]. Supporting people to conduct safe and dignified burials (SDB) is, accordingly, a recognised pillar of EVD epidemic responses [18].

Evidence on the effect of SDB on the propagation of EVD epidemics is very limited, but suggests that the intervention could reduce transmission considerably [19]. Its implementation in West Africa, however, was marked by poor adherence to protocols and a biosafety-driven approach exemplified by excluding families from performing funerary rites or observing burials; bodies being buried or cremated without the consent or knowledge of the family; and the use of unmarked or mass graves. This approach contributed to low trust and uptake of the service [20, 21]. An SDB service should instead implement strict infection prevention and control while simultaneously preserving the dignity of the deceased and enabling families and communities to participate in funerary and burial rites in a culturally acceptable way [17, 22]. In strictly epidemiological terms, the effect of SDB on transmission should be a function of its coverage (whether SDB takes place at all), timeliness (when different components of SDB take place, relative to the infectiousness of a deceased person over time), whether different components of the service (see below) are actually implemented, and the expected reduction in transmissibility parameters including the contact rate and probability of transmission per contact, that taken together determine the reproduction number. A mathematical treatment of these relationships is proposed in Additional file 1.

During the 2018–2020 eastern DRC epidemic, the DRC Red Cross Society, supported by the International Federation of the Red Cross and Red Crescent Societies (IFRC), was charged with implementing and coordinating the SDB service. The SDB package included community engagement, a safe and dignified burial at an appropriate site agreed with the family, modifications to traditional funerary customs, psychosocial support, EBOV testing of the deceased and decontamination of dwellings. While the Red Cross coordinated provision of this package, psychosocial support was provided by the Danish Refugee Council and Unicef, and testing by the Ministry of Health and WHO. SDB activities were undertaken by mobile rapid reaction teams localised in various response hubs. To facilitate community acceptance, the service was embedded within a broader community engagement strategy and continuously adapted based on community feedback [22]. Initially, the mobile teams were staffed by Red Cross volunteers only. However, limited access to particularly insecure communities, combined with an aspiration to empower government and civil society actors, soon prompted the establishment of local ‘Civil Protection’ teams (operational from 23 September 2018), which performed the largest share of SDBs (see below). Civil Protection teams operated similarly to Red Cross SDB teams, but were staffed by health workers designated and overseen by rural or urban *commune* (administrative level 3) governments. From 12 December 2018, a community-led emergency harm reduction burial (CEHRB) scheme was also operational, whereby local community members, supported with training and equipment by the Red Cross or Civil Protection, performed burials whenever SDB mobile teams could not reach the location on time. A key difference between CEHRB and other SDB teams was that the former were static rather than mobile, working within their communities of residence and resupplied from neighbouring health facilities.

Our study aim was to evaluate the performance of the Red Cross-supported SDB service during the 2018–2020 EVD epidemic in eastern DRC in order to inform improvements for future outbreaks. Specifically, we wished to (i) assess the performance and implementation fidelity of SDB activities against pre-specified service standards and (ii) identify factors affecting the performance of the SDB intervention. To do this, we did a retrospective cohort study of SDB instances documented by the IFRC, with data on potential factors associated with SDB performance drawn from various external sources in addition to the SDB dataset itself. In a separate paper (submitted), we estimate the effect of the SDB service on EBOV transmission.

## Methods

### Study period and population

The geographic scope and timeframe of analysis were North Kivu, Ituri and South Kivu provinces (population ≈ 6,300,000) from 6 August 2018 (date of first recorded SDB dispatch alert) until 10 October 2019, comprising about 91% of all confirmed or probable EVD cases during the epidemic. All SDB alerts received during this period were eligible for analysis.

### Description of the SDB service

**Fig. 1:**
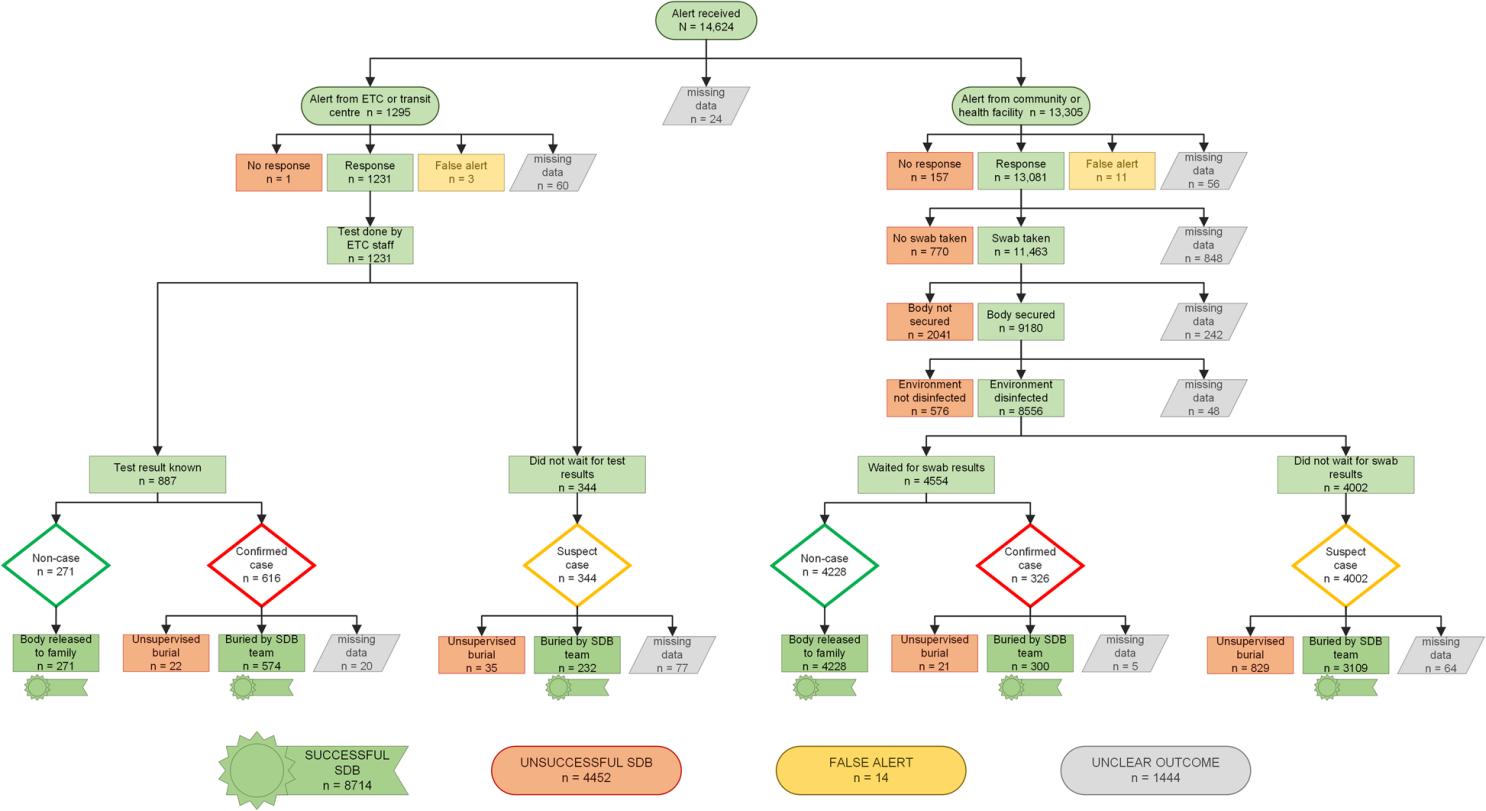
Flowchart of SDB alerts, by response outcome and EVD status

Figure 1 represents the intended SDB process as a flowchart. Once a death was reported, the ‘Alerts System’ was activated. For EBOV-positive deaths in an Ebola treatment centre (ETC), a dispatch alert was sent directly to the SDB team whereas for suspected cases in the community and in other health facilities, the MoH and WHO Case Investigation Team was first notified in order to determine whether the decedent met the case definition of a suspected EVD case (Additional file 1, Table S1). If the decedent was determined to be a suspect case, a dispatch alert was sent to the closest SDB sub-coordination hub (geographical base within which different SDB teams were based), who dispatched a team to the decedent’s location. The team’s expected composition is detailed in Additional file 1, Table S2.

Once on site, the SDB team was expected to perform the following core tasks: collection of an oral swab specimen for EBOV testing, if not already taken; securing of the body by disinfection with 0.5% chlorine and placement in a leak-proof body bag; decontamination of the dwelling where the deceased resided with 0.5% chlorine; decontamination of the deceased’s belongings with 0.5% chlorine (if not possible, these were to be destroyed safely or placed in the grave); and burial of the body at a site agreed with relatives in a grave at least two metres deep covered by at least one and a half metres of earth to protect it from wild animals. For suspected cases, SDB was to be performed prior to obtaining EBOV test results if consent was provided by the family; if not, the body was to be taken to a mortuary to await results. During burial, family representatives were invited to join the SDB team in securing the body. In order to improve acceptance and inclusivity, the Red Cross employed community engagement and accountability personnel who trained volunteers and liaised with community and religious leaders as well as the family to assist with any feasible modifications to the standard SDB. These staff also analysed community feedback information to identify improvements to SDB services (the use of community feedback information generated by the Red Cross during the epidemic is the subject of a separate paper [6]).

### Quantifying SDB performance

The IFRC maintained an individual, standardised Excel database of all SDB ‘alerts’ received (i.e. requests by a community, a health facility or other actors to deploy a team to assist with burial): this database recorded details of the decedent’s demographic profile, dates (day, time) of alert and completion of different SDB steps, whether key activities in the SDB package were successfully accomplished, and reasons for unsuccessful/incomplete burial deployments. Because the dataset does not distinguish mobile from static (CEHRB) SDBs supported by Civil Protection teams, our analysis henceforth considers three SDB actors: (i) Red Cross mobile teams, (ii) Red Cross-supported CEHRB and (iii) Civil Protection mobile or CEHRB teams.

After applying range and consistency checks, we analysed the SDB database to compute a set of key performance indicators of intervention fidelity and performance (Table 1). The targets in Table 1 were set by the Red Cross team as aspirational benchmarks of success and were not tied to quantitative epidemiological control requirements.

**Table 1:** Key performance indicators for evaluating SDB performance

Key performance indicator	Indicator type	Target^a^ (%)	Definition
Proportion of dispatch alerts responded to by an SDB team	Process	100	A ‘dispatch alert’ is the final confirmatory alert that an SDB is required (the death is a suspected or confirmed EVD case and the family have consented for an SDB to take place). ‘Responded’ is defined as an SDB team being deployed to the location with the intention of following the SDB process.
Proportion of swabs performed	Process	100	For community deaths only: collection of an oral swab specimen to be sent to the laboratory for EVD testing.
Proportion of corpses secured	Process	100	For community deaths only: the corpse (except for the face) is sprayed with 0.5% chlorine and placed inside a disinfected, leakproof, opaque body bag.
Proportion of dwellings disinfected	Process	100	For community deaths only: the dwelling and environment where the deceased resided are disinfected by spraying with 0.5% chlorine. Soiled items and mattresses/bed mats are burnt (to be replaced at a later date). The deceased’s infected belongings are collected in a disinfected plastic bag to be buried with the body.
Proportion of dispatch alerts in which there was community and/or nonacceptance	Process	not defined	Community and/or family nonacceptance is defined as either the community or the family declining the SDB, or any behaviour from the community or the family to disrupt the SDB process. This includes withholding consent for an SDB, verbal or physical aggression or violence against the SDB team, occultation of the corpse and removal of the corpse from the body bag.
Proportion of EVD suspected/confirmed deaths for which safe burials were conducted	Output	80	A ‘safe burial’ is defined as: ▪ for ETC deaths: reception of a secured body and burial in a grave by, or supervised by, an SDB team. ▪ for community (including deaths in other health facilities) deaths: Collection of an oral swab specimen, securing of the body, disinfection of the environment, and burial in a grave by, or supervised by, an SDB team.
Proportion of dispatch alerts successfully responded to by an SDB team	Output	80	A ‘successful response’ is defined as: ▪ safe burial performed for confirmed cases with EVD status known on SDB team arrival; or ▪ body secured while testing results are pending, with safe burial performed if the EVD test comes back positive or release of the body to the family if the EVD test comes back negative; or ▪ safe burial performed for suspect cases with no EVD test result or for whom it is unfeasible to wait for test results An ‘unsuccessful response’ is defined as: ▪ an SDB team did not respond to the dispatch alert ▪ a safe burial was not performed for a confirmed or suspected EVD death
Proportion of SDB responses completed within 24 h from time of alert	Output	80	Proportion of all SDB responses (successful and unsuccessful) for which the recorded time of dispatch alert and recorded end/time are within 24 h of each other.
Proportion of SDB responses completed within 72 h from time of alert	Output	100	Proportion of all SDB responses (successful and unsuccessful) for which the recorded time of dispatch alert and recorded end/time are within 72 h of each other.

^a^As set by the SDB programme

### Risk factor analysis

We developed a causal framework of domains and factors potentially determining SDB performance (Additional file 1, Fig. S1). The framework was based on the authors’ knowledge of EVD and associated response interventions, the context and published literature. We then sourced data that would capture as many of the domains in the framework as possible. Most explanatory variables were sourced from key humanitarian websites and EVD response actors in DRC, but some (e.g. lack of community acceptance) were contained within the SDB dataset itself. Explanatory and population datasets we identified are described in Additional file 1 (Table S3): we classified these within (i) distal (gender; age; density of health facilities; road network; mining activity); (ii) intermediate (stage of the epidemic locally; recent EVD incidence; deaths due to insecurity events; attacks against the EVD response; suspensions of EVD services; availability of a nearby EVD treatment centre; number of phone networks; number of radio frequencies); and (iii) proximal (type of SDB team; setting of death; community and/or family nonacceptance of the SDB) levels of causality. Where appropriate, we divided the variable of interest by population to compute a rate and created categories out of continuous variables. We expected that some of the variables would mainly function as proxies of the causal domains of interest, rather than direct measures: for example, mining activity may capture differences in overall economic livelihood as well as movement patterns, in turn affecting EBOV transmission.

We modelled the binomial probability of unsuccessful SDB with a logit link function, defined as per Table 1, as a function of any of the above explanatory variables. We fitted a generalised linear mixed model of the log odds of SDB failure, specifying the sub-coordination hub as a random effect, since we assumed observations within each hub to be correlated. We firstly computed univariate associations, screening out variables if they had an association with *p*-value ≥ 0.20. We next fitted a model composed of the remaining distal variables, retaining any that were significant at *p*-value < 0.05, heavily influenced the coefficient of any of the other associations or improved the model’s goodness-of-fit based on the Akaike Information Criterion values of models with and without the variable; we then added intermediate level variables, retaining variables based on the above rules, and lastly proximal variables. We considered potential effect modifications but none were evident. We also observed model diagnostics including the distribution of residuals and their correlation with observations and fitted values. As this was an observational study, we include the Strengthening the Reporting of OBservational studies in Epidemiology (STROBE) checklist [23] in Additional file 1.

## Results

### Output of the SDB service

**Fig. 2:**
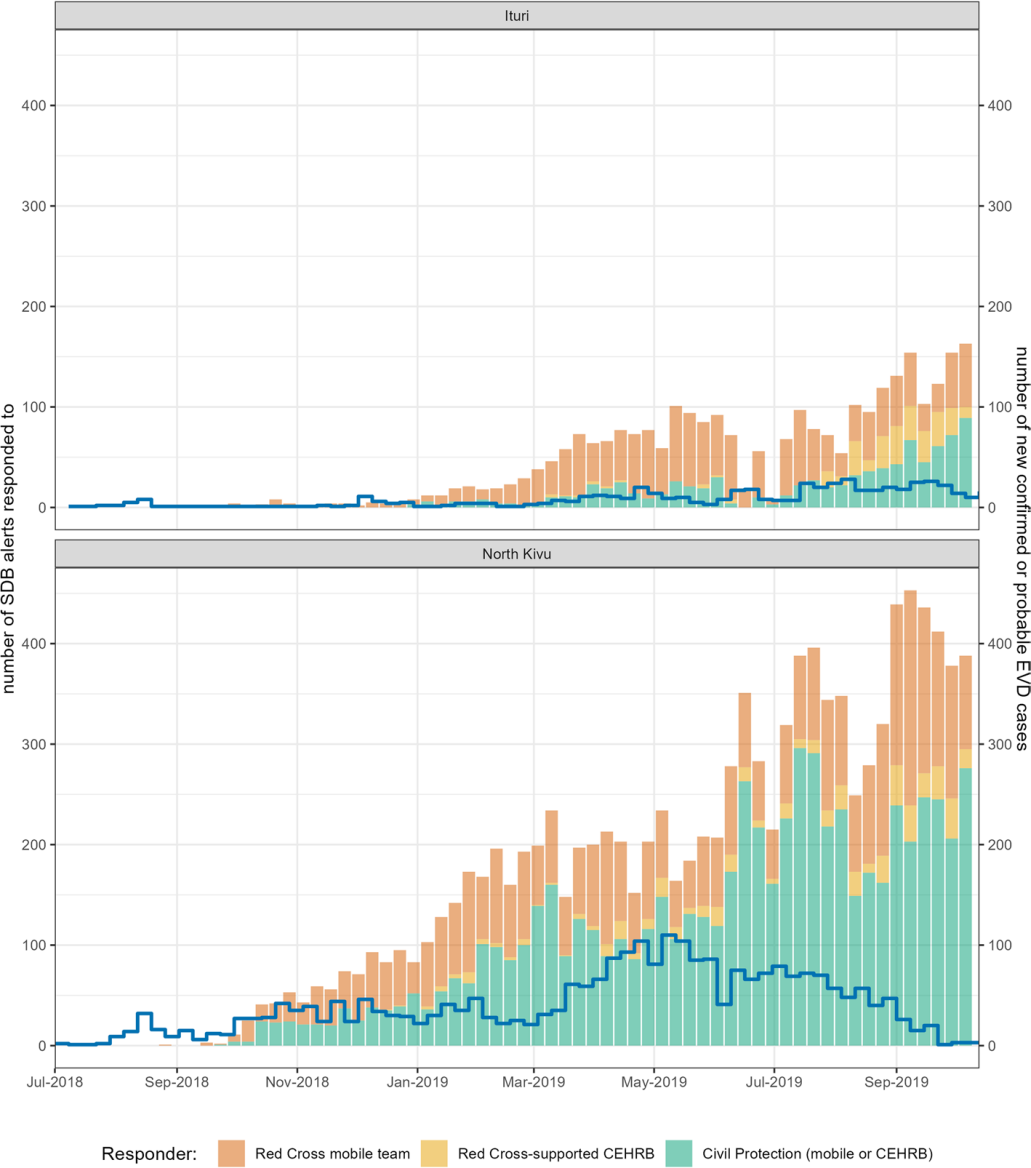
Trends in incident confirmed and probable EVD cases (blue line) and SDB alerts responded to (bars) by type of team, week and province. CEHRB, Community Emergency Harm Reduction Burial static teams

A total of 14,624 dispatch alerts were recorded in the Red Cross SDB database between 6 August 2018 and 10 October 2019 (Table 2). Of these, 79% originated from North Kivu, 21% from Ituri and 0.01% from South Kivu. Beni health zone experienced the highest number of alerts (2851, 20%), followed by Katwa (1592, 11%), Bunia (1,251, 9%) and Butembo (1216, 8%); see Additional file 1, Table S4 for data by health zone. ETCs and patient transit centres accounted for 9% of all alerts (1,160 and 135, respectively), while the majority of alerts were from community settings, including health facilities, households and other sites. SDB responses increased in volume throughout the epidemic period, even as reported cases declined (Fig. 2).

**Table 2:** Characteristics of decedents for whom an SDB alert was raised

Variable	EBOV status (rapid test or PCR result)		
	Negative (%)	Positive (%)	Unknown^a^ (%)
Gender			
Female	2793 (43.7)	543 (54.1)	3116 (43.1)
Male	3534 (55.3)	454 (45.3)	3752 (51.9)
Unknown/missing	60 (0.9)	6 (0.6)	366 (5.1)
Age			
< 1 year old	1195 (18.7)	49 (4.9)	1628 (22.5)
1–4 years old	579 (9.1)	95 (9.5)	728 (10.1)
5–17 years old	492 (7.7)	122 (12.2)	612 (8.5)
18–59 years old	2269 (35.5)	618 (61.6)	2401 (33.2)
≥ 60 years old	1524 (23.9)	103 (10.3)	1398 (19.3)
Unknown/missing	328 (5.1)	16 (1.6)	467 (6.5)
Setting of death			
Community	2653 (41.5)	191 (19.0)	3409 (47.1)
ETC	272 (4.3)	613 (61.1)	410 (5.7)
Hospital/health facility	3462 (54.2)	199 (19.8)	3391 (46.9)
Unknown/missing	0 (0.0)	0 (0.0)	24 (0.3)
Sub-coordination hub from which the SDB team responded			
Beni	987 (15.5)	296 (29.5)	3227 (44.6)
Bunia	1156 (18.1)	3 (0.3)	328 (4.5)
Butembo	2744 (43.0)	604 (60.2)	1363 (18.8)
Goma	886 (13.9)	1 (0.1)	352 (4.9)
Komanda	30 (0.5)	12 (1.2)	964 (13.3)
Mandima	304 (4.8)	63 (6.3)	717 (9.9)
Other	277 (4.3)	23 (2.3)	281 (3.9)
Unknown/missing	3 (0.0)	1 (0.1)	2 (0.0)
Totals	6387 (43.7)	1003 (6.9)	7234 (49.5)

^a^Unknown EBOV status typically means that the result of the test carried out on the sample collected during the SDB was not fed back to the SDB coordination team, meaning that the database instance could not be updated with a positive or negative result

About half of responses (*n* = 7627, 53%) were conducted by Civil Protection teams, including mobile or static CEHRB (Fig. 2). Among the 6755 SDBs supported by the Red Cross, the CEHRB approach was adopted for 841 alerts (13%). Overall, 52% (7740) of alerts were for male decedents. More than half of alerts (57%) were for individuals aged ≥ 18 years old but a considerable number were for infants (Table 2). Only 7% (1003) of alerts featured a positive EBOV test result.

### Performance of the SDB service

Table 3 summarises key performance indicators by setting of death and EVD status. Excluding false alerts, 1% (161) of dispatch alerts were recorded as not responded to by either a mobile or CEHRB team. The main recorded reasons for these instances of non-response were community and/or family nonacceptance (39%) and insecurity (32%). Logistical issues (21%) included no team being available (8%), a lack of supply materials (body bags, coffins or personal protective equipment) and/or transport (8%), and inaccessibility of locations (5%).

**Table 3:** Values of key performance indicators for different components of the SDB response, by category of deceased person

Indicator type	Key performance indicator	Target	Category of deceased				
			All SDB alerts	Confirmed and suspect cases	Confirmed cases	Suspected cases	Non-cases
Community and ETC deaths			(*n* = 14,610)	(*n* = 8247)	(*n* = 995)	(*n* = 7252)	(*n* = 6363)
Process	Proportion of dispatch alerts responded to by an SDB team	100%	98.9% (14,313/14,474)				
Process	Proportion of dispatch alerts in which there was community and/or family nonacceptance	undefined	12.5% (1754/14,035)	18.6% (1435/7726)	4.8% (48/991)	20.6% (1387/6735)	5.1% (319/6309)
Output	Proportion of dispatch alerts for which safe burials were conducted	80%	57.1% (7549/13,218)	60.7% (4215/6950)	90.5% 874/966	55.8% (3341/5984)	53.2% (3334/6268)
Output	Proportion of SDB responses completed within 24 h from time of alert	80%	88.9% (11,290/12,700)	88.6% (6261/7069)	82.8% (775/936)	89.5% (5486/6133)	89.3% (5029/5631)
Output	Proportion of SDB responses completed within 72 h from time of alert	100%	99.9% (12,684/12,700)	99.9% (7063/7069)	99.8% (934/936)	99.9% (6129/6133)	99.8% (5621/5631)
Output	Proportion of dispatch alerts successfully responded to by an SDB team	80%	66.2% (8,714/13,166)				
Community deaths only			(*n* = 13,294)	(*n* = 7202)	(*n* = 378)	(*n* = 6824)	(*n* = 6092)
Process	Proportion of swabs collected	100%	93.6% (11,469/12,260)				
Process	Proportion of corpses secured	100%	77.5% (9893/12,761)	77.3% (5179/6689)	94.1% (352/374)	76.4% (4827/6315)	77.6% (4714/6072)
Process	Proportion of dwellings disinfected	100%	76.1% (9844/12,931)	78.4% (5376/6855)	86.9% (338/389)	77.9% (5045/6478)	73.5% (4468/6076)

Community and/or family nonacceptance once the team actually arrived on site was recorded for 13% of SDB responses. The proportion of suspected or confirmed EVD deaths receiving SDBs (61%) was lower than the 80% target. However, this target was surpassed (91%) if only confirmed cases are considered.

Considering community responses only, collection of oral swabs was near target at 94% completion; securing of the body and disinfection of dwellings featured lower completion (78% and 76% for suspected and confirmed cases, respectively, increasing to 94% and 88% if considering EBOV+ deaths only). Recorded reasons for SDB failure at community level included, as above, community and/or family nonacceptance, insecurity and logistical issues (data not shown).

**Fig. 3:**
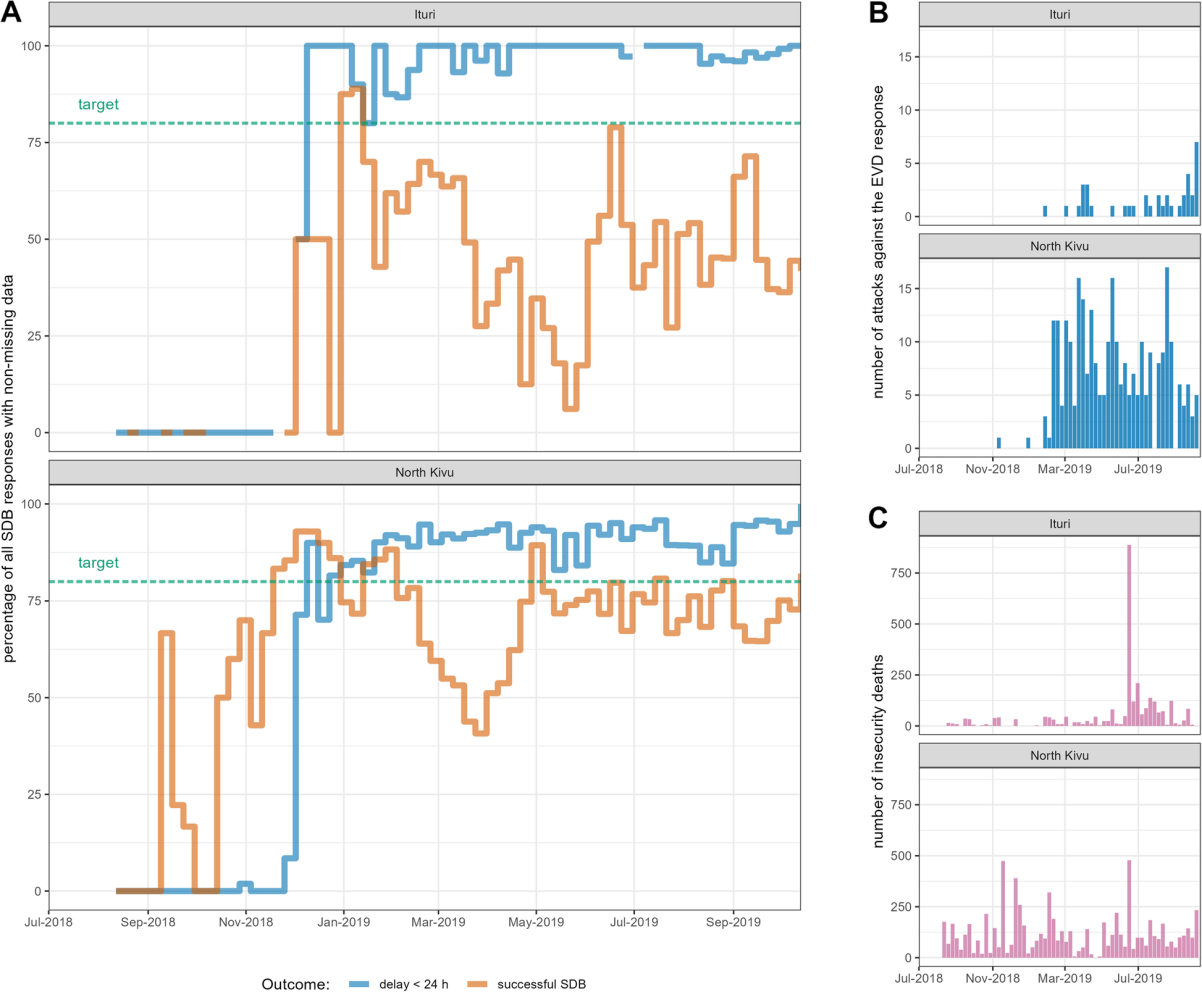
**A** Percentages of successful (orange line) and timely (within 24 hours; blue line) SDB responses, by province and week. **B** Number of attacks against the EVD response, by province and week. **C** Number of deaths directly resulting from recorded insecurity incidents (other than attacks against the EVD response), by province and week

In terms of timing, 89% of SDB responses were completed within 24 h, exceeding the target of 80%, while almost all (99.9%) were completed within 72 h. In both Ituri and North Kivu, from December 2018 onwards the proportion of timely SDBs increased, but both provinces experienced large monthly fluctuations in the proportion of successful SDBs throughout 2019 (Fig. 3). Overall, the proportion of all alerts successfully responded to (66%) fell short of the 80% target.

### Risk factors for unsuccessful SDB

A total of 17 variables ranging from proximal to distal were assessed for their association with SDB failure, 12 of which were carried into multivariate analysis. The final model (Table 4) included the following explanatory variables: gender, number of health facilities per population, stage in the epidemic, presence of any confirmed EVD cases during the previous 6 weeks, occurrence of any suspensions in the EVD response during the previous 6 weeks, number of deaths due to insecurity events per population during the previous 2 weeks, presence of an operational EVD treatment centre, number of mobile phone networks, number of radio frequencies with reception, type of SDB team, occurrence of community and/or family nonacceptance of the SDB and setting in which the death occurred.

**Table 4:** Univariate and multivariate associations between explanatory variables and the odds of SDB failure. Odds ratios (ORs) below 1 indicate lower odds than the reference category, and vice versa

Variable	Univariate analysis			Multivariate analysis		
	OR	95% CI	*p*-value	OR	95% CI	*p*-value
Distal level factors						
Age of the decedent						
18 to 59 years	[1.00]	-		[1.00]	-	
0 years	0.93	0.83 to 1.03	0.172	0.91	0.79 to 1.05	0.203
1 to 4 years	0.83	0.71 to 0.97	0.016	0.73	0.60 to 0.89	0.002
5 to 17 years	1.02	0.88 to 1.19	0.786	0.91	0.75 to 1.11	0.345
≥ 60 years	1.18	1.06 to 1.31	0.002	1.04	0.92 to 1.19	0.531
Gender of the decedent						
Male	[1.00]	-		[1.00]	-	
Female	0.92	0.85 to 1.00	0.047	0.93	0.84 to 1.03	0.158
Number of health facilities per 100,000 population within the health zone						
≥ 50.0	[1.00]	-		[1.00]	-	
25.0 to 49.9	1.26	1.09 to 1.44	0.001	1.21	0.98 to 1.49	0.070
< 25.0	2.33	2.11 to 2.58	< 0.001	1.57	1.07 to 2.30	0.021
Road length per 100,000 population within the health zone						
≥ 400 km	[1.00]	-				
200 to 399 km	0.89	0.66 to 1.19	0.427			
< 200 km	0.36	0.27 to 0.48	< 0.001			
Mining activity within the health zone						
No mining	[1.00]	-				
Some mining	0.43	0.38 to 0.49	< 0.001			
Intermediate level factors						
Stage in the epidemic within the health zone						
No confirmed cases yet	[1.00]	-		[1.00]	-	
During epidemic	0.95	0.71 to 1.27	0.737	2.14	1.35 to 3.38	0.001
Post-epidemic	1.29	0.98 to 1.69	0.069	3.60	2.32 to 5.58	< 0.001
Any confirmed EVD cases during the previous 6 weeks within the health zone						
No	[1.00]	-		[1.00]	-	
Yes	0.59	0.52 to 0.67	< 0.001	0.49	0.40 to 0.60	< 0.001
Attacks against the EVD response during the previous 6 weeks within the health zone						
No	[1.00]	-				
Yes	1.01	0.92 to 1.11	0.798			
Any suspension of EVD response activities during the previous 6 weeks within the health zone						
No	[1.00]	-		[1.00]	-	
Yes	0.78	0.72 to 0.85	< 0.001	1.61	1.43 to 1.82	< 0.001
Number of deaths due to insecurity events per 100,000 population occurring during the previous 2 weeks within the district (*territoire*)						
0	[1.00]	-		[1.00]	-	
> 0	1.53	1.38 to 1.71	< 0.001	1.73	1.47 to 2.03	< 0.001
Presence of an operational EVD treatment centre or transit/isolation centre within the health zone						
Yes	[1.00]	-		[1.00]	-	
No	2.30	2.09 to 2.52	< 0.001	3.59	2.92 to 4.42	< 0.001
Number of mobile phone networks with reception within the health zone						
< 4	[1.00]	-		[1.00]	-	
4	0.43	0.39 to 0.47	< 0.001	1.53	1.10 to 2.12	0.011
Number of radio frequencies with reception within the health zone						
< 10	[1.00]	-		[1.00]	-	
10 to 19	0.44	0.38 to 0.52	< 0.001	3.61	2.51 to 5.21	< 0.001
≥ 20	0.87	0.76 to 0.99	0.032	7.43	5.77 to 9.58	< 0.001
Proximal level factors						
SDB team type						
Red Cross mobile	[1.00]	-		[1.00]	-	
static CEHRB, Red Cross supported	0.68	0.55 to 0.85	< 0.001	0.25	0.18 to 0.35	< 0.001
Civil Protection (mobile or static CEHRB)	1.22	1.11 to 1.33	< 0.001	0.61	0.54 to 0.70	< 0.001
Community and/or family nonacceptance of the SDB						
No	[1.00]	-		[1.00]	-	
Yes	37.64	31.19 to 45.42	< 0.001	40.20	32.06 to 50.39	< 0.001
Setting in which the death occurred						
EVD treatment centre or transit/isolation centre	[1.00]	-		[1.00]	-	
Hospital	7.68	5.83 to 10.12	< 0.001	7.84	5.73 to 10.73	< 0.001
Community	12.16	9.22 to 16.03	< 0.001	11.18	8.15 to 15.34	< 0.001

At the distal level, reduced health facility coverage per population was associated with higher odds of SDB failure (odds ratio [OR] 1.57, 95% confidence interval [95%CI] 1.07 to 2.30 comparing the lowest- and highest-coverage HZs; *p* = 0.021). At the intermediate level, odds of failure were higher when the local epidemic was ongoing (OR 2.14, 95%CI 1.35 to 3.38; *p* = 0.001) or ended (OR 3.60, 95%CI 2.32 to 5.58, *p* < 0.001), compared to pre-epidemic (namely the period before the first ascertained EVD case in a given HZ, during which the SDB service responded only to alerts for decedents who were EBOV- or had unknown infection status); however, the local occurrence of confirmed cases in the previous month and a half was associated with about half the odds of failure (OR 0.49, 95%CI 0.40 to 0.60; *p* < 0.001). Suspensions of EVD response activities, as well as insecurity, were associated with 1.61 (95%CI 1.43 to 1.82; *p* < 0.001) and 1.73 (OR 1.47 to 2.03; *p* < 0.001) times higher odds of failure respectively, as were the presence of an EVD treatment facility locally (OR 3.59, 95%CI 2.92 to 4.42, *p* < 0.001), and a high coverage of mobile telephony (OR 1.53, 95%CI 1.10 to 2.12; *p* = 0.011) and radio (OR 7.43, 95%CI 5.77 to 9.58, comparing the highest- and lowest-coverage HZs, *p* < 0.001). At the proximal level, SDBs carried out by Red Cross-supported local community members under the static CEHRB programme or mainly supported by Civil Protection teams featured 0.25 (95%CI 0.18 to 0.35; *p* < 0.001) and 0.61 (95%CI 0.54 to 0.70; *p* < 0.001) times lower odds of failure than other SDBs, respectively. Any community and/or family nonacceptance was strongly associated with SDB failure (OR 40.20, 95%CI 32.06 to 50.39; *p* < 0.001). Lastly, SDBs for deaths in hospitals and in communities had markedly higher odds of failure (7.84, 95%CI 5.73 to 10.73; *p* < 0.001 and 11.18, 95%CI 8.15 to 15.34; *p* < 0.001, respectively) compared to deaths in EVD treatment or isolation centres.

We also fitted a model to SDB data restricted to known EBOV + decedents (*n* = 1003; see Additional file 1, Table S5). Some variables, including epidemic stage and SDB team type, were not featured in this model due to data sparsity. The associations with communication means (phone and radio network availability) were not evident in this model, but strong associations of SDB failure with EVD response suspensions, community and/or family nonacceptance and setting of death remained.

## Discussion

### Main findings

The DRC remains affected by crisis conditions. During the main epidemic year of 2018, 1.8 million people were displaced by violence and 12.8 million were in need of humanitarian assistance [24]. With > 100 active armed groups, the eastern DRC is a challenging environment in which to conduct epidemic responses [8]. In such a context, the National Red Cross Society, supported by the IFRC, can leverage its large volunteer network and mandate to support epidemic-affected communities. The Red Cross-led SDB service became available within the first days of the epidemic declaration and achieved a remarkable volume of activity over the 14-month period we analysed. The service’s scale-up was however somewhat delayed, potentially missing opportunities to contain transmission clusters during the first 2–3 months of the outbreak. Conversely, towards the end of the epidemic, the introduction of more specific case definitions or criteria for requesting SDB might have improved efficiency.

SDB teams almost ubiquitously responded to alerts and met timeliness targets. The proportion of successful SDB responses was below target, though low performance was mainly circumscribed to a period from February to May 2019: this period saw high-profile killings of EVD responders, attacks against treatment centres [25, 26] and increased insecurity in health zones reporting EBOV transmission, particularly in Ituri [27], as shown in Fig. 3. Direct threats against Red Cross international staff also occurred during this period, resulting in temporary evacuations from North Kivu. Performance was also lower when SDB teams responded to suspected, rather than confirmed EVD deaths. Despite these reductions in service performance, we find in a separate paper (in preparation) that SDB was associated with substantial reductions in EBOV transmission.

We identified notable risk factors for SDB failure. Plausibly, local insecurity and interruptions to the EVD response were associated with higher odds of failure. Insecurity constrained the EVD response generally and, specifically, reduced SDB access to communities [28]. Burial supported by Civil Protection teams (mobile or CEHRB), and/or by CEHRB teams supported by the Red Cross, was associated with a lower odds of failure, suggesting the benefit of empowering local authorities and localising services to affected communities themselves (note that the true proportion of CEHRB SDBs includes an unknown number supported by Civil Protection). Strong community engagement has been shown to contribute to the effectiveness of EVD response in the West African epidemic [29, 30]. On the other hand, Civil Protection teams, unlike the Red Cross, sometimes travelled with armed escorts and may have contributed to ‘militarising’ the EVD response, with consequences for trust and acceptance [31].

Generally, SDB teams encountered limited community and/or family nonacceptance, but its occurrence was associated with a very high odds of failure, as noted in the West Africa epidemic and more broadly for EVD response interventions in DRC [32–35]. In the DRC, the Red Cross has engaged with affected communities through a Community Engagement and Accountability approach [36], whereby community volunteers routinely collect information regarding local knowledge, attitudes and practices in order to systematically respond to needs and concerns and adapt EVD response programming to meet communities’ expressed needs. These volunteers accompany SDB teams, liaising with families, community and religious leaders to safely adjust burial procedures based on local customs and preferences. In West Africa, a similar model of mediation, where implemented, increased SDB acceptance [37].

We found that the presence of communication infrastructure (radio, mobile network), as well as community exposure to EVD (health zones being in the midst or past their epidemic; a nearby EVD treatment centre) were associated with higher SDB failure odds. Communication means should in theory facilitate dissemination of response information and healthy behaviours [38]. In this scenario, however, increased communication may have facilitated the spread of negative views of the EVD response, information on inappropriate practices among responders, or conspiracy theories. The presence of communication infrastructure may have had a particularly marked effect once the epidemic had reached a given HZ, explaining why pre-epidemic SDBs were more successful: however, this effect modification was not evident statistically. Moreover, the above risk factors may be proxies rather than direct measures of the infrastructure causal domain. Generally, our and other studies’ findings need to be interpreted with reference to a context of chronic disenfranchisement and inadequate provision of health and other essential services, which time-bound, outbreak-focussed community engagement activities might not sufficiently address [8].

### Study limitations

This was a retrospective evaluation, featuring no direct observation of SDB activities and relying principally on monitoring data not originally collected for research purposes. While we observed no patterns suggesting data fabrication, some SDB teams may have felt an incentive to overstate successful activities. Instances of CEHRB burial were anecdotally subject to greater-than-average data completeness and timeliness problems, which may have resulted in under-reporting of failed or untimely alert responses, and thus an over-estimation of the programme’s performance. The SDB dataset contained some ambiguities. EVD status was not ascertained or communicated to the SDB database for a large proportion of deaths in the community: these decedents were considered suspect cases and thus included in our analysis, but some may in fact not have met the suspect case definition, potentially biasing our estimates of key performance indicators. Findings could have been strengthened through qualitative exploration of the perceptions of EVD-affected communities and SDB staff. Separately, we are analysing community feedback data collected by Red Cross volunteers to shed light on this. Lastly, our risk factor analysis was limited by the explanatory and confounder variables that we were able to source: the model would have been strengthened by other variables for which we were unable to find appropriate and complete data: hidden confounding may therefore be present in our analysis. Specifically, the extent to which community engagement and feedback during specific SDB instances were used to adapt the service locally or generally could have been an important factor behind SDB success, and would have thus confounded the other associations. Separately the limited quality of data may have introduced random or non-random error (the former is likely, and would generally bias estimates of association towards non-significance). Generally, exploratory risk factor models with multiple adjustment are subject to the well-described ‘Table 2 fallacy’ [39], and should be interpreted with caution so as, in this case, identify factors that should be evaluated further and considered carefully in future interventions.

While we were able to analyse SDB performance when the service was called upon, we were unable to quantify its coverage (proportion of EBOV+ decedents for whom a SDB was performed). The coverage numerator (EBOV+ SDB burials) is unclear due to the many SDB instances for which no EBOV serostatus was recorded in the database after sample collection (Table 3). The denominator contains an unknown number of EVD deaths that escaped case ascertainment (at least one report [40] suggests a substantial fraction of undetected cases). Because burial at the community level mostly could not be delayed while awaiting results of EBOV testing, the SDB service targeted all decedents that community members may have suspected as EVD cases, meaning about four times as many SDBs were performed as all known EVD cases in the epidemic. If a crude measure of coverage is adopted, whereby *any* decedent during the epidemic should have received SDB, then over the entire population of HZs affected by the epidemic over the 14 months of the analysis period, and assuming a crude annual death rate of about 10 per 1000 for DRC [41], some 66,000 deaths would have been expected, meaning SDB coverage would have been ≈ 20%: this is likely to be a gross underestimate of the true coverage, as it is plausible that true EVD cases would have been much more likely than at random to have been reached by the SDB service, since the latter was only triggered by cases meeting the EVD suspect definition.

Generally, our study evaluates only one aspect of the SDB service and omits dimensions such as its feasibility, fidelity to different components, acceptability, equity and cost-effectiveness, some of which would be illuminated by a more comprehensive implementation science approach to evaluation adopting epidemiological, social science and health economics methods [42].

### Conclusions

This evaluation was enabled by collection of systematic, quality monitoring data on SDBs by the IFRC and partners, reinforcing the benefit of such programmatic data collection. Our evidence shows that a large-scale, timely and moderately performant SDB service was feasible despite the challenging circumstances of the EVD response in eastern DRC. Failed SDBs due to the inability to secure the body or supervise burials suggest weaknesses in community engagement. The considerable number of SDBs for which EVD status remained unclear indicates a gap in testing or reporting of results. Community and/or family nonacceptance, while infrequent, is a key barrier to SDB effectiveness: an acceptance approach emphasising cooperation and with communities and other stakeholders should be pursued [17, 43]. Critically, the apparent success of localised SDB implemented by static teams within affected communities (the CEHRB programme) suggests this model should be relied upon and documented further in future EVD epidemics, particularly where accessibility is a constraint for mobile teams. Its potential benefits, however, should be carefully weighed against risks (insufficient burial safety, infection of SDB performers and family members). Lastly, our findings suggest that the availability of communication means and access to news may not necessarily improve community sentiment about epidemic responses, and trust in control interventions or their implementing actors: as noted by others [8, 34], solutions to this are likely to involve a better understanding of how information flows, engagement with communities through consultative processes based on active social listening, and empowering people themselves to be the main bearers of helpful knowledge.

### Supplementary Information

**Additional file 1.** Appendix containing (i) the STROBE checklist for cohort observational study reports; (ii) A mathematical expression of the theoretical effect of SDB on EBOV transmission; (iii) Additional figures and tables: Figure S1 and Tables S1-S5. **Fig S1.** Causal framework of factors potentially affecting SDB performance. **Table S1.** Case definitions adopted by the World Health Organisation during the EVD epidemic. **Table S2.** Expected composition of a SDB team. **Table S3.** Data sources for explanatory variables. **Table S4.** Values of selected key performance indicators for different components of the SDB response, by category of deceased person and location. **Table S5.** Univariate and multivariate associations between explanatory variables and the odds of SDB failure, restricting analysis only to SDBs with EBOV+ decedent status (*n* = 1003).

## Data Availability

All source data and R analysis scripts are available at https://github.com/francescochecchi/evd_drc_sdb_performance.

## Abbreviations

CEHRB: Community-led emergency harm reduction burial
CI: Confidence interval
DRC: Democratic Republic of the Congo
EBOV: Ebola virus
ETC: Ebola treatment centre
EVD: Ebola virus disease
IFRC: International Federation of Red Cross and Red Crescent Societies
OR: Odds ratio
SDB: Safe and dignified burial
WHO: World Health Organisation
