# Supplementary material for: Performance of a safe and dignified burial intervention during an Ebola epidemic in the eastern Democratic Republic of the Congo, 2018–2019
Source: BMC Med. 2023 Dec 5;21:484. doi: 10.1186/s12916-023-03194-x (PMC10696665; doi:10.1186/s12916-023-03194-x)
Supplement: Supplementary file 1 — Additional file 1. Appendix containing (i) the STROBE checklist for cohort observational study reports; (ii) A mathematical expression of the theoretical effect of SDB on EBOV transmission; (iii) Additional figures and tables: Figure S1 and Tables S1-S5. Fig S1. Causal framework of factors potentially affecting SDB performance. Table S1. Case definitions adopted by the World Health Organisation during the EVD epidemic. Table S2. Expected composition of a SDB team. Table S3. Data sources for explanatory variables. Table S4. Values of selected key performance indicators for different components of the SDB response, by category of deceased person and location. Table S5. Univariate and multivariate associations between explanatory variables and the odds of SDB failure, restricting analysis only to SDBs with EBOV+ decedent status (n = 1003). [file 12916_2023_3194_MOESM1_ESM.docx]

Performance of a safe and dignified burial intervention during an Ebola epidemic in the eastern Democratic Republic of the Congo, 2018-2019

ADDITIONAL FILE 1

# STROBE Checklist for cohort studies

|  | Item No | Recommendation | Reported? (section) |
| --- | --- | --- | --- |
| **Title and abstract** | 1 | (*a*) Indicate the study’s design with a commonly used term in the title or the abstract | Yes (Abstract) |
|  |  | (*b*) Provide in the abstract an informative and balanced summary of what was done and what was found | Yes (Abstract) |
| Introduction | | |  |
| Background/rationale | 2 | Explain the scientific background and rationale for the investigation being reported | Yes (Background) |
| Objectives | 3 | State specific objectives, including any prespecified hypotheses | Yes (end of Background) |
| Methods | | |  |
| Study design | 4 | Present key elements of study design early in the paper | Yes (end of Background) |
| Setting | 5 | Describe the setting, locations, and relevant dates, including periods of recruitment, exposure, follow-up, and data collection | Yes (Methods, Study period and population) |
| Participants | 6 | (*a*) Give the eligibility criteria, and the sources and methods of selection of participants. Describe methods of follow-up | Yes for eligibility criteria (Methods, Study period and population)  No for methods of follow-up (no follow-up visits done) |
|  |  | (*b*) For matched studies, give matching criteria and number of exposed and unexposed | No – not a feature of the study |
| Variables | 7 | Clearly define all outcomes, exposures, predictors, potential confounders, and effect modifiers. Give diagnostic criteria, if applicable | Yes (Methods, Quantifying SDB performance and Risk factor analysis) |
| Data sources/ measurement | 8* | For each variable of interest, give sources of data and details of methods of assessment (measurement). Describe comparability of assessment methods if there is more than one group | Yes for sources of data (Additional file 1)  No for comparability of assessment methods – not relevant |
| Bias | 9 | Describe any efforts to address potential sources of bias | Yes (Methods, Risk factor analysis) |
| Study size | 10 | Explain how the study size was arrived at | No – exhaustive study |
| Quantitative variables | 11 | Explain how quantitative variables were handled in the analyses. If applicable, describe which groupings were chosen and why | Yes (Methods, Quantifying SDB performance and Risk factor analysis) |
| Statistical methods | 12 | (*a*) Describe all statistical methods, including those used to control for confounding | Yes (Methods, Quantifying SDB performance and Risk factor analysis) |
|  |  | (*b*) Describe any methods used to examine subgroups and interactions | No – not a feature of this study |
|  |  | (*c*) Explain how missing data were addressed | Yes (Methods, Quantifying SDB performance and Risk factor analysis) |
|  |  | (*d*) If applicable, explain how loss to follow-up was addressed | No – not a feature of this study |
|  |  | (*e*) Describe any sensitivity analyses | Yes (Results, Risk factors for unsuccessful SDB) |
| Results | | |  |
| Participants | 13* | (a) Report numbers of individuals at each stage of study—eg numbers potentially eligible, examined for eligibility, confirmed eligible, included in the study, completing follow-up, and analysed | Yes (Results, Output of the SDB service) |
|  |  | (b) Give reasons for non-participation at each stage | Yes (Results, Output of the SDB service) |
|  |  | (c) Consider use of a flow diagram | Yes (Results, Output of the SDB service, Figure 2) |
| Descriptive data | 14* | (a) Give characteristics of study participants (eg demographic, clinical, social) and information on exposures and potential confounders | Yes (Results, Output of the SDB service, Table 2) |
|  |  | (b) Indicate number of participants with missing data for each variable of interest | Yes (Results, Output of the SDB service, Figure 2) |
|  |  | (c) Summarise follow-up time (eg, average and total amount) | No – not a feature of this study |
| Outcome data | 15* | Report numbers of outcome events or summary measures over time | Yes (Results, Performance of the SDB service) |
| Main results | 16 | (*a*) Give unadjusted estimates and, if applicable, confounder-adjusted estimates and their precision (eg, 95% confidence interval). Make clear which confounders were adjusted for and why they were included | Yes (Results, Risk factors for unsuccessful SDB) |
|  |  | (*b*) Report category boundaries when continuous variables were categorized | Yes (Results, Risk factors for unsuccessful SDB) |
|  |  | (*c*) If relevant, consider translating estimates of relative risk into absolute risk for a meaningful time period | No – not relevant |
| Other analyses | 17 | Report other analyses done—eg analyses of subgroups and interactions, and sensitivity analyses | Yes (Results, Risk factors for unsuccessful SDB) |
| Discussion | | |  |
| Key results | 18 | Summarise key results with reference to study objectives | Yes (Discussion, Main findings) |
| Limitations | 19 | Discuss limitations of the study, taking into account sources of potential bias or imprecision. Discuss both direction and magnitude of any potential bias | Yes (Discussion, Study limitations) |
| Interpretation | 20 | Give a cautious overall interpretation of results considering objectives, limitations, multiplicity of analyses, results from similar studies, and other relevant evidence | Yes (Discussion, Main findings, Study limitations) |
| Generalisability | 21 | Discuss the generalisability (external validity) of the study results | Yes (Discussion, Study limitations) |
| Other information | | |  |
| Funding | 22 | Give the source of funding and the role of the funders for the present study and, if applicable, for the original study on which the present article is based | Yes (Declarations) |

# Theoretical effect of safe and dignified burials on Ebola virus transmission

Let $R_{0}$ be the basic reproduction number of Ebola virus (EBOV), i.e. the mean cumulative number of infections arising from a single infected individual in a fully susceptible population. We may define $R_{0}$ as the sum of the contributions to onward transmission made when the individual is infected but still alive, dead due to infection (for the proportion of infections that do result in death) or alternatively a recovered survivor of infection:

$$R_{0}=R_{0,l}+R_{0,d}+R_{0,s}$$

where $l$ = alive, $d$ = dead and $s$ = survivor.

The above quantities can be broken down as follows:

$$R_{0}=\int_{t_{0}}^{t_{\text{max},l}} c_{t}\beta_{t}+\mu\int_{t_{d}}^{t_{\text{max},d}} c_{t}\beta_{t}+(1-\mu)\int_{t_{s}}^{t_{\text{max},s}} c_{t}\beta_{t}$$

where $t_{0}$ and $t_{\text{max},l}$ are respectively the start and end of the period of infectiousness in people who are infected but still alive; $\mu$ is the infection-fatality ratio; $t_{d}$ and $t_{\text{max},d}$are the time of death and the maximum time at which a dead body will still be infectious; $t_{s}$ and $t_{\text{max},s}$are the time of recovery from infection in survivors and the maximum time at which survivors will still be infectious; $c_{t}$ is the number of contacts the infectious individual has with susceptible people during time interval $t$ (these may be direct, like transfer of body fluids, or indirect, like contact with contaminated fomites); and $\beta_{t}$ is the probability that transmission will occur per contact, which may itself vary over time, for example as a function of when certain symptoms such as haemorrhage, diarrhoea or vomiting are more likely to occur. All quantities are averages and assumed to reflect conditions without any control interventions.

The safe and dignified burial (SDB) package implemented during the 2018-2020 DRC epidemic consists of several components, including decontaminating the body, placing the body in a body bag, decontaminating the living quarters, disposing of the decedent’s clothes and finally burying the body in a safe way. Each of these $K$ components may be thought of as affecting one of the possible routes of transmission whereby an EBOV+ dead body may infect susceptible people, thereby contributing to an overall relative effect $\varphi$ (between 0 and 1) of SDB on $R_{0,d}$. Accordingly, we can write

$$\varphi=1-\int_{t_{d}}^{t_{\text{max},d}} max\left( 0,\sum_{k=1}^{k=K} \delta_{k,t}\left( {(1-\varphi}_{k,c})c_{t}(1-\varphi_{k,\beta})\beta_{t} \right) \right)$$

Here, for each time increment, we sum the effect of all $K$ SDB components, which is in turn a function of $\delta_{k,t}$, an indicator variable of coverage ($0$ = the component has not yet been implemented; $1$ = the component has been implemented), $\varphi_{k,c}$ is the reduction in the contact rate $c_{t}$ expected from component $k$ and $\varphi_{k,\beta}$ is the corresponding reduction in the probability of transmission per contact $\beta_{t}$ . We constrain the total sum of component effects to $\leq0$, recognising that some components (e.g. securing the body, burial) may be epidemiologically redundant.

In the above formulation, coverage, timeliness and success (i.e. full implementation) of SDB, as defined in the main text, all contribute to $\delta_{k,t}$ by (i) increasing the fraction of a dead body’s infectiousness period that is mitigated by SDB interventions and (ii) decreasing the effective contact rate during this fraction of time. Note that the effect of timeliness may not be linear, since much depends on *when* dead bodies are most infectiousness: for example, if the SDB team arrives after the maximum acceptable time that family members are willing to wait before proceeding with funeral practices themselves, most of the opportunity for reducing transmission will be lost.

Lastly, the relative effect of SDB on overall transmissibility will be $\frac{R_{0}-\varphi R_{0,d}}{R_{0}}$ . An implication is that, if $\left( R_{0}-\varphi R_{0,d} \right)<1$, SDB alone in the absence of other control interventions could potentially drive the epidemic towards extinction. This outcome however would only be possible if $\left( R_{0}-R_{0,d} \right)<1$ , i.e. if transmissibility while alive or a survivor only sums up to < 1.0 onward infections; furthermore, it would presuppose very high SDB coverage, timeliness and completeness.

# Additional figures and tables


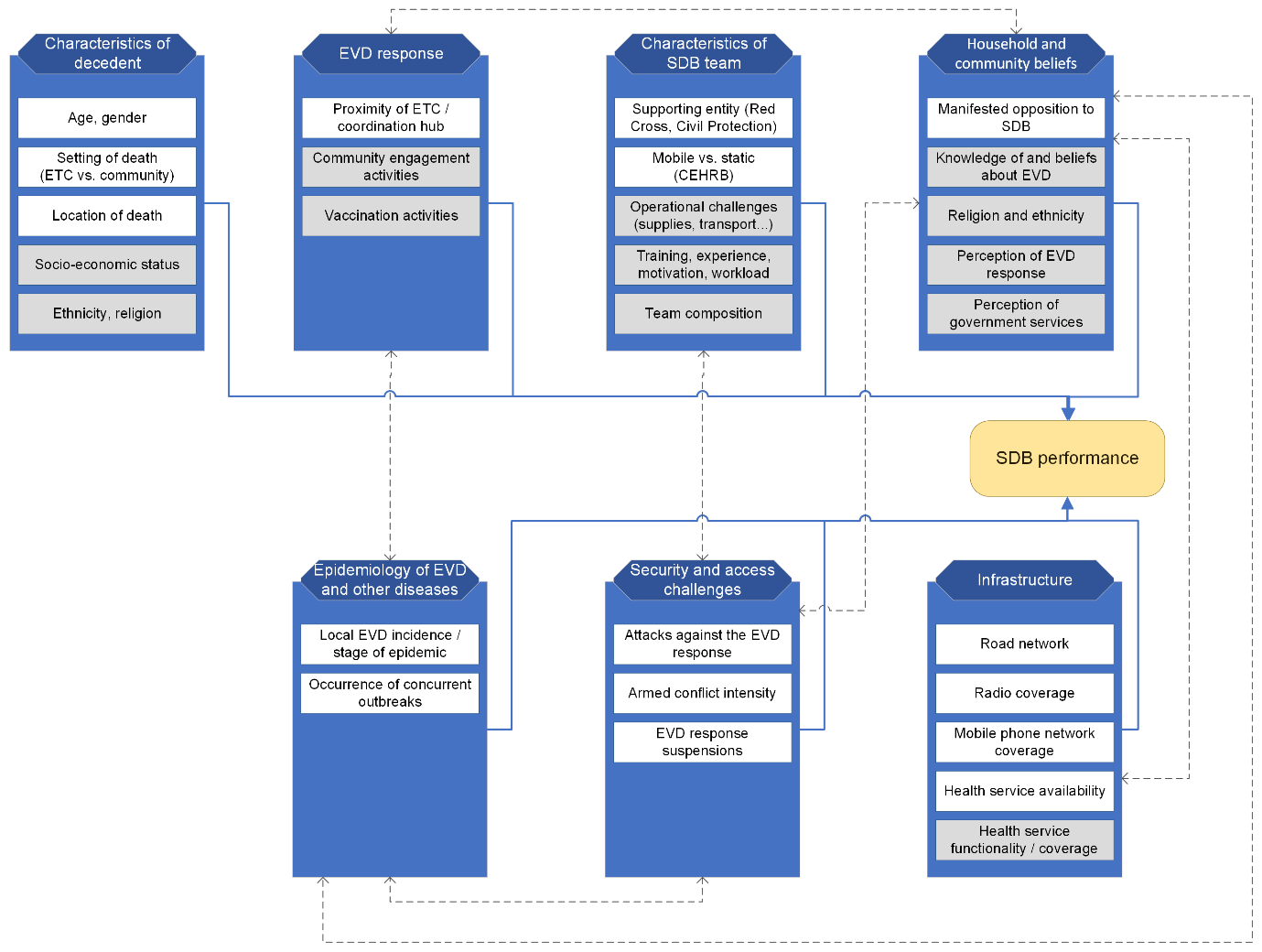


Figure S1. Causal framework of factors potentially affecting SDB performance. Expected feedback loops between different domains are shown as grey dashed arrows.

Table S1. Case definitions adopted by the World Health Organisation during the EVD epidemic†.

| Classification | Definition |
| --- | --- |
| Suspected case | a. Any person, alive or dead, suffering or having suffered from a sudden onset of high fever and having had contact with:  - a suspected, probable or confirmed Ebola case;  - a dead or sick animal; OR  b. Any person with sudden onset of high fever and at least three of the following symptoms:  - headaches  - lethargy  - anorexia / loss of appetite  - aching muscles or joints  - stomach pain  - difficulty swallowing  - vomiting  - difficulty breathing  - diarrhoea  - hiccups; OR  c. Any person with inexplicable bleeding; OR  d. Any sudden, inexplicable death |
| Probable case | a. Any suspected case evaluated by a clinician; OR  b. Any deceased suspected case (where it has not been possible to collect specimens for laboratory confirmation) having an epidemiological link with a confirmed case  (Note: if laboratory specimens are collected in due time during the illness, the preceding categories are reclassified as “laboratory confirmed” cases and “non-case”.) |
| Laboratory confirmed case | Any suspected or probable cases with a positive laboratory result. Laboratory confirmed cases must test positive for the virus antigen, either by detection of virus RNA by reverse transcriptase-polymerase chain reaction (RT- PCR), or by detection of IgM antibodies directed against Ebola. |
| Non-case | Any suspected or probable case with a negative laboratory result. “Non-case” showed no specific antibodies, RNA or specific detectable antigens. |

†Source: World Health Organization. Case definition recommendations for Ebola or Marburg virus diseases. Interim Guideline. 2014 (<https://apps.who.int/iris/handle/10665/146397>).

Table S2. Expected composition of a SDB team.

| Job role | Number | Brief description |
| --- | --- | --- |
| Team leader/technical supervisor | 1 | Overall coordination of the activity, supervision and quality assurance |
| Body handler | 4 | Collects oral swab specimen, handles the body to place the body in body bag and transport the secured remains, full PPE |
| Hygienist: type 1 | 1 | Sprayer (0.5% chlorine), in full PPE, to disinfect the body and body bag, and fomites in the areas used by the deceased |
| Hygienist: Type 2 | 1 | Sprayer (0.5% chlorine), light PPE†, to assist with donning and doffing of full PPE. |
| Hygienist: Type 3 | 2-4 | Disinfection of materials, vehicles, etc. and management of reusable materials at the SDB base. Do not participate in securing or burying the deceased. |
| Community engagement person | 1 | Liaison with community and family to discuss options for safe burial, collection of feedback data |
| Driver | 2 |  |
| Psychosocial support | 1, if available | (provided by another actor) |

† Light PPE = surgical gown, gloves, mask, goggles.

Table S3. Data sources for explanatory variables.

| Source | Causal domain | Data elements carried into analysis |
| --- | --- | --- |
| The Armed Conflict Location & Event Data Project  (ACLED)  (<https://acleddata.com/tag/democratic-republic-of-congo/>)  The ACLED project relies on local and international media sources, as well as networks of civil society informants, to passively collect information on a range of insecurity events: these are, to the extent possible, dated, geolocated and characterised according to a common dictionary of metavariables, including the number of deaths arising from the event. | Security and access challenges | Number of insecurity incidents and deaths of any typology per *territoire* (district, comprising several health zones) during the previous 2 epidemiological weeks, excepting any attacks against the EVD response. |
| World Health Organisation (WHO)  [not public]  Throughout the response, the WHO maintained a line list of individual attacks or insecurity incidents affecting the Ebola response, with details for each including dates, locations and specific impacts, including whether, where and for how long the incident resulted in suspension of response activities. | Security and access challenges | Number of attacks against/insecurity incidents involving personnel or facilities involved in the EVD response, per health zone and epidemiological week. |
|  |  | [derived variable:] Whether EVD response activities were suspended in any given health zone at any point during the previous 6 epidemiological weeks. |
| World Health Organisation (WHO, 2019) (<https://www.afro.who.int/health-topics/ebola-disease/situation-reports>)  Narrative situation reports on the Ebola response. | EVD response | Presence of an EVD treatment centre (including holding / triage centres) in a given health zone and epidemiological week. |
| Ministry of Health and WHO (2020)  (<https://data.humdata.org/dataset/ebola-cases-and-deaths-drc-north-kivu>)  This aggregate dataset was derived from the official line list of EVD cases in the epidemic. | Epidemiology of EVD and other diseases | Number of new EVD cases by status (confirmed, probable and suspected), per health zone and epidemiological week. |
|  |  | [derived variable:] Exposure to the epidemic of a given health zone per epidemiological week (pre-epidemic, during epidemic, post-epidemic). |
|  |  | [derived variable:] Whether any EVD cases occurred in the health zone over the previous 6 weeks (as a marker for recent transmission) |
| International Federation of the Red Cross and Red Crescent Societies (IFRC, 2018-2019)  [not public]  SDB dataset, as described in the main text (Methods). | Characteristics of decedent | Gender, age and EBOV serostatus (positive, negative, unknown / unclear) of the decedent. |
|  |  | Whether the decedent was located at an ETC, at a hospital or in the community when the SDB alert was raised. |
|  | Characteristics of SDB team | Whether the SDB alert was responded to by a Red Cross mobile team, a Red Cross-supported static CEHRB team or a Civil Protection team (mobile or static / CEHRB). |
|  | Household and community beliefs | Whether community and/or family nonacceptance of the SDB was reported. |
| World Health Organization (2018)  (<https://data.humdata.org/dataset/drc-health-facilities>)  Geolocated dataset of public primary and secondary health facilities, developed during the epidemic for improved situational awareness. | Infrastructure | Number and type of health facilities per health zone as of 2018 (note that the dataset did not contain details on the functionality of each facility). |
| Novetta (2018)  [not public]  Novetta is a private company contracted to supply information services for the Ebola response, as part of which it monitored radio and public social media content. So as to support this work, the company mapped the availability of radio and telephony across the epidemic-affected region. | Infrastructure | Number of phone networks with reliable coverage per health zone, as of 2018. |
|  |  | Number of radio stations with reliable signal per health zone, as of 2018. |
| United Nations World Food Programme (2019)  (<https://data.humdata.org/dataset/democratic-republic-of-the-congo-road-network-main-roads>)  Geospatial dataset of DRC’s road network, which we aggregated to health zone level. | Infrastructure | Total Km of road network per health zone. |
| International Peace Information Service (<https://data.humdata.org/dataset/cod_mines_curated_all_opendata_p_ipis>)  Dataset from an initiative to map informal mining sites across Eastern DRC ever active from 2009 to 2019 inclusive. | Infrastructure | Number of mining sites per health zone. |

Table S4. Values of selected key performance indicators for different components of the SDB response, by category of deceased person and location.

| Key performance  indicator | Province | Sub-coordination hub | Category of deceased | | | |
| --- | --- | --- | --- | --- | --- | --- |
|  |  |  | Suspected and confirmed cases | | Confirmed cases | |
|  |  |  | Total cases | Percentage | Total cases | Percentage |
| Community and ETC deaths | | | | | | |
| Proportion of suspected/ confirmed EVD deaths for which safe burials were conducted | Ituri | Biakato | 194 | 64.8% (92/142) | 15 | 76.9% (10/13) |
|  |  | Bunia | 329 | 7.7% (22/286) | 3 | 100.0% (2/2) |
|  |  | Komanda | 976 | 76.5% (551/720) | 12 | 83.3% (10/12) |
|  |  | Mambasa | 29 | 33.3% (8/24) | 2 | 100.0% (1/1) |
|  |  | Mandima | 780 | 77.2 (318/412) | 63 | 97.9% (46/47) |
|  | North Kivu | Beni | 3,526 | 59.3% (1,898/3,200) | 297 | 88.1% (258/293) |
|  |  | Butembo | 1,976 | 67.1% (1,212/1,807) | 595 | 91.4% (540/591) |
|  |  | Goma | 353 | 26.3% (85/323) | 1 | 100.0% (1/1) |
|  |  | Mangina | 79 | 78.1 (25/32) | 5 | 100.0% (4/4) |
|  | South Kivu | Mwenga | 1 | 100.0% (1/1) | 1 | 100.0% (1/1) |
| Proportion of SDB responses completed within 24 hours of dispatch alert | Ituri | Biakato | 391 | 92.9% (327/352) | 15 | 73.3% (11/15) |
|  |  | Bunia | 1,484 | 98.7% (606/614) | 3 | 100.0% (3/3) |
|  |  | Komanda | 1,006 | 98.8% (894/905) | 12 | 90.9% (10/11) |
|  |  | Mambasa | 78 | 98.5% (64/65) | 2 | 100.0% (2/2) |
|  |  | Mandima | 1,084 | 68.7% (699/1,018) | 63 | 60.0% (36/60) |
|  | North Kivu | Beni | 4,499 | 78.0% (3,065/3,931) | 297 | 55.9% (139/249) |
|  |  | Butembo | 4,711 | 96.8% (4,343/4,487) | 595 | 96.6% (568/588) |
|  |  | Goma | 1,239 | 99.1% (1,210/1,221) | 1 | 100.0% (1/1) |
|  |  | Mangina | 110 | 76.2% (77/101) | 5 | 60% (3/5) |
|  | South Kivu | Mwenga | 1 | 100.0% (1/1) | 1 | 100.0% (1/1) |
| Community deaths only | | | | | | |
| Proportion of corpses secured | Ituri | Biakato | 389 | 87.2% (321/368) | 14 | 85.7% (12/14) |
|  |  | Bunia | 1,479 | 36.9% (528/1,431) | 2 | 100.0% (2/2) |
|  |  | Komanda | 982 | 83.2% (789/948) | 7 | 85.7% (6/7) |
|  |  | Mambasa | 75 | 71.8% (51/71) | 0 | - |
|  |  | Mandima | 896 | 91.8% (679/740) | 11 | 90.9% (10/11) |
|  | North Kivu | Beni | 3,975 | 87.0% (3,290/3,783) | 81 | 97.4% (75/77) |
|  |  | Butembo | 4,155 | 72.0% (2,955/4,103) | 257 | 93.8 (241/257) |
|  |  | Goma | 1,232 | 97.3% (1,194/1,227) | 0 | - |
|  |  | Mangina | 105 | 96.4% (81/84) | 5 | 100.0% (5/5) |
|  | South Kivu | Mwenga | 1 | 100.0% (1/1) | 1 | 100.0% (1/1) |

Table S5. Univariate and multivariate associations between explanatory variables and the odds of SDB failure, restricting analysis only to SDBs with EBOV+ decedent status (n = 1003). Odds ratios (ORs) below 1 indicate lower odds than the reference category, and vice versa.

| Variable | Univariate analysis | | | Multivariate analysis | | |
| --- | --- | --- | --- | --- | --- | --- |
|  | OR | 95%CI | p-value | OR | 95%CI | p-value |
| Distal level factors | | | | | | |
| Age of the decedent | | | | | | |
| 18 to 59y | [1.00] | - |  | [1.00] | - |  |
| 0y | 0.78 | 0.27 to 2.25 | 0.642 | 0.95 | 0.28 to 3.22 | 0.938 |
| 1 to 4y | 0.67 | 0.30 to 1.50 | 0.326 | 0.62 | 0.25 to 1.52 | 0.298 |
| 5 to 17y | 0.51 | 0.23 to 1.15 | 0.103 | 0.55 | 0.23 to 1.28 | 0.165 |
| ≥ 60y | 0.90 | 0.44 to 1.83 | 0.775 | 0.88 | 0.39 to 1.97 | 0.750 |
| Gender of the decedent | | | | | | |
| Male | [1.00] | - |  |  | - |  |
| Female | 0.77 | 0.51 to 1.18 | 0.228 |  |  |  |
| Number of health facilities per 100,000 population within the health zone | | | | | | |
| ≥ 50.0 | [1.00] | - |  | [1.00] | - |  |
| 25.0 to 49.9 | 0.51 | 0.30 to 0.87 | 0.013 | 0.63 | 0.31 to 1.28 | 0.203 |
| < 25.0 | 2.13 | 0.84 to 5.43 | 0.112 | 2.11 | 0.68 to 6.59 | 0.198 |
| Road length per 100,000 population within the health zone | | | | | | |
| ≥ 400 Km | [1.00] | - |  |  |  |  |
| 200 to 399 Km | 1.97 | 0.64 to 6.06 | 0.235 |  |  |  |
| < 200 Km | 1.10 | 0.49 to 2.47 | 0.819 |  |  |  |
| Mining activity within the health zone | | | | | | |
| no mining | [1.00] | - |  |  |  |  |
| some mining | 1.22 | 0.78 to 1.91 | 0.384 |  |  |  |
| Intermediate level factors | | | | | | |
| Stage in the epidemic within the health zone | | | | | | |
| no confirmed cases yet | [1.00] | - |  |  |  |  |
| during epidemic | n/a (singular fit problem) | | | | | |
| post-epidemic | n/a (singular fit problem) | | | | | |
| Any confirmed EVD cases during the previous 6 weeks within the health zone | | | | | | |
| No | [1.00] | - |  |  |  |  |
| Yes | n/a (singular fit problem) | | | | | |
| Attacks against the EVD response during the previous 6 weeks within the health zone | | | | | | |
| No | [1.00] | - |  |  |  |  |
| Yes | 1.34 | 0.75 to 2.38 | 0.317 |  |  |  |
| Any suspension of EVD response activities during the previous 6 weeks within the health zone | | | | | | |
| No | [1.00] | - |  | [1.00] | - |  |
| Yes | 1.76 | 1.04 to 2.97 | 0.034 | 4.70 | 2.34 to 9.43 | < 0.001 |
| Number of deaths due to insecurity events per 100,000 population occurring during the previous 2 weeks within the district (*territoire*) | | | | | | |
| 0 | [1.00] | - |  |  |  |  |
| > 0 | 1.46 | 0.92 to 2.33 | 0.108 |  |  |  |
| Presence of an operational EVD treatment centre or transit / isolation centre within the health zone | | | | | | |
| Yes | [1.00] | - |  |  |  |  |
| No | 1.93 | 0.91 to 4.08 | 0.086 |  |  |  |
| Number of mobile phone networks with reception within the health zone | | | | | | |
| < 4 | [1.00] | - |  |  |  |  |
| 4 | 0.45 | 0.24 to 0.85 | 0.013 |  |  |  |
| Number of radio frequencies with reception within the health zone | | | | | | |
| < 10 | [1.00] | - |  |  |  |  |
| 10 to 19 | 1.22 | 0.54 to 2.74 | 0.636 |  |  |  |
| ≥ 20 | 0.98 | 0.45 to 2.13 | 0.950 |  |  |  |
| Proximal level factors | | | | | | |
| SDB team type | | | | | | |
| Red Cross mobile | [1.00] | - |  |  |  |  |
| static CEHRB, Red Cross supported | 1.19 | 0.40 to 3.52 | 0.750 |  |  |  |
| Civil Protection (mobile or static CEHRB) | 1.04 | 0.68 to 1.60 | 0.858 |  |  |  |
| Community and/or family nonacceptance of the SDB | | | | | | |
| No | [1.00] | - |  | [1.00] | - |  |
| Yes | 15.35 | 8.04 to 29.31 | < 0.001 | 16.02 | 7.26 to 35.36 | < 0.001 |
| Setting in which the death occurred | | | | | | |
| EVD treatment centre or transit/isolation centre | [1.00] | - |  | [1.00] | - |  |
| hospital | 4.96 | 2.75 to 8.93 | < 0.001 | 5.20 | 2.67 to 10.14 | < 0.001 |
| community | 9.34 | 5.33 to 16.36 | < 0.001 | 8.64 | 4.50 to 16.59 | < 0.001 |
